# PPAR*α* Ligands as Antitumorigenic and Antiangiogenic Agents

**DOI:** 10.1155/2008/906542

**Published:** 2008-08-14

**Authors:** Ambra Pozzi, Jorge H. Capdevila

**Affiliations:** Department of Medicine, Division of Nephrology and Hypertension, S-3223 Medical Center North, Vanderbilt University, Nashville, TN 37232, USA

## Abstract

Peroxisome proliferator-activated receptors (PPARs) belong to the nuclear receptor family of ligand-activated transcription factors. This subfamily is composed of three members—PPAR*α*, PPAR*δ*, and PPAR*γ*—that differ in their cell and tissue distribution as well as in their target genes. PPAR*α* is abundantly expressed in liver, brown adipose tissue, kidney, intestine, heart, and skeletal muscle; and its ligands have been used to treat diseases such as obesity and diabetes. The recent finding that members of the PPAR family, including the PPAR*α*, are expressed by tumor and endothelial cells together with the observation that PPAR ligands regulate cell growth, survival, migration, and invasion, suggested that PPARs also play a role in cancer. In this review, we focus on the contribution of PPAR*α* to tumor and endothelial cell functions and provide compelling evidence that PPAR*α* can be viewed as a new class of ligand activated tumor “suppressor” gene with antiangiogenic and antitumorigenic activities. Given that PPAR ligands are currently used in medicine as hypolipidemic drugs with excellent tolerance and limited toxicity, PPAR*α* activation might offer a novel and potentially low-toxic approach for the treatment of tumor-associated angiogenesis and cancer.

## 1. THE PEROXISOMAL PROLIFERATOR-ACTIVATED RECEPTORS (PPARs)

PPARs nuclear receptors that regulate many physiological processes, including lipid and glucose homeostasis, inflammation, and wound healing [1]. Three PPAR isotypes have been identified: *α*, *δ* (or *β*), and *γ*. Upon ligand binding, PPARs form heterodimers with the retinoic acid receptor and interact with specific response elements in the promoter region of target genes [2]. Although PPARs share extensive structural homology, each isotype appears to possess distinct functions. PPAR*γ* is expressed mainly in adipose tissue and at lower levels in intestine and immune cells [3–5]. It controls adipocyte differentiation, glucose and lipid homeostasis [5–7] and has been implicated in the pathophysiology of insulin resistance and atherosclerosis [1, 8]. PPAR*γ* ligands include long-chain fatty acids, prostaglandins, and other eicosanoids [4]. Among the synthetic PPAR*γ* ligands, the thiazolidinediones are currently used as insulin sensitizers in patients with type-2 diabetes [9]. PPAR*δ* is ubiquitously expressed and it is most abundant in brain, colon, and skin [10, 11], and binds molecules such as fatty acids and prostaglandins [4].

PPAR*α* is primarily expressed in liver, brown adipose tissue, kidney, intestine, heart, and skeletal muscle. This receptor controls fatty acid metabolism and transport, peroxisomal and mitochondrial *β*-oxidation [3, 4]. Moreover, this receptor has been implicated in the pathophysiology of inflammation and cardiovascular diseases [12]. Several compounds bind PPAR*α*, including fatty and phytanic acids [4], as well as the fibric acid derivatives used in medicine for the treatment of hyperlipidemias [1].

## 2. PPARs AND CANCER

The observation that members of the PPAR family are expressed by tumor and endothelial cells [13, 14] together with the finding that PPAR ligands regulate cell growth, survival, migration, and invasion [15, 16] prompted investigators to determine whether these receptors play a role in the pathophysiology of tumorigenesis and angiogenesis [17, 18].

The anticancer effects of PPAR*γ* agonists have been extensively studied because of their antiproliferative, proapoptotic, antiapoptotic, and differentiation-promoting activities [19]. In this context, activation of PPAR*γ* has been reported to reduce tumor cell proliferation and invasion [20] and to enhance apoptosis [21]. PPAR*γ* ligands also regulate endothelial cell growth, migration, and angiogenesis [22–25], and influence the progression of vascular inflammation and tumorigenesis [26, 27]. Moreover, disruption of the PPAR*γ* gene in the intestine enhances tumorigenesis in Apc^Min/+^ mice [28]. Although these studies suggest that PPAR*γ* functions as a tumor suppressor factor and its activation might be beneficial for patients with tumors, PPAR*γ* agonists have been shown also to increase the frequency of colon tumors [29] and to promote edema [30].

In contrast to PPAR*γ*, PPAR*δ* has been described as protumorigenic as its ligand-mediated activation increases tumor-associated angiogenesis [31]. Moreover, treatment of Apc^Min/+^ mice with PPAR*δ* antagonists or crossing these mice with PPAR*δ*-null mice prevents tumor growth and angiogenesis [31]. However, a recent study showed that activation of this receptor attenuates chemically-induced colon carcinogenesis, and that PPAR*δ*-null mice exhibit increased colon polyp multiplicity, suggesting that ligand activation of this receptor can also inhibit carcinogenesis [32].

The analysis of the antitumorigenic properties of PPAR*α* ligands has been less studied mostly due to the observation that long-term administration of certain PPAR*α* agonists (Clofibrateand WY14643) induces hepatocarcinogenesis in rodents [33–35], despite the fact that PPAR*α* ligands are widely used in medicine as antilipidemic drugs with excellent tolerance and little or no reported side effects. The finding that fenofibrate decreases VEGF levels in patients with hyperlipidemiaand atherosclerosis [36] provided a rationale for analyzing PPAR*α* and its ligands as a molecular target for cancer therapy. In this review, we highlight some of the key functions attributed to PPAR*α* in the context of endothelial and tumor cell biology.

## 3. PPAR*α* TARGETS IN ANGIOGENESIS

**Figure 1:**
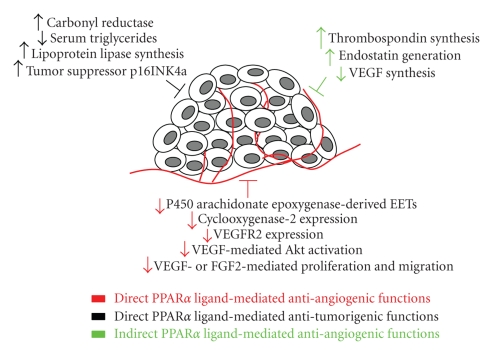
* Schematic representation of the antiangiogenic and antitumorigenic properties of PPAR*α**. PPAR*α* ligands reduce tumor growth by direct inhibition of tumor cell functions (black pathway). In addition, they prevent tumor-associated angiogenesis via direct (red pathway) as well as indirect (green pathway) inhibition of endothelial cell functions.

PPAR*α* controls the transcription of many genes involved in cell functions such as lipid metabolisms, inflammation, cell cycle progression, and angiogenesis. Among the angiogenic targets, PPAR*α* has been shown to regulate the expression of the vascular endothelial growth factor (VEGF), fibroblast growth factors (FGFs), members of the arachidonic acid P450 monooxygenases, thrombospondin and endostatin to name few (see also Figure 1 and Table 1). Biscetti et al. have recently shown that the selective PPAR*α* agonist WY14643 promotes cornea angiogenesis in vivo and enhances endothelial tubulogenesis in vitro [37]. Interestingly, WY14643 can enhance endothelial cell tubulogenesis in vitro only when endothelial cells are cocultured with interstitial cells and this effect is accompanied by upregulation of interstitial-derived VEGF synthesis [37]. However, WY14643 does not directly promote endothelial cell migration or proliferation, and when used at 10–20 *μ*M range it reduces both endothelial cell proliferation and migration [37]. Thus, this study indicates that while WY14643 might directly prevent endothelial cell functions, it might also promote angiogenesis by stimulating the production of nonendothelial VEGF. The observation that activation of PPAR*α* prevents endothelial cell proliferation/migration parallels our findings that WY14643 prevents—in a PPAR*α*-dependent fashion—endothelial cell proliferation in vitro and tumorigenesis in vivo [38]. The antiangiogenic properties of WY14643 are associated with a PPAR*α*-dependent downregulation of the epoxygenase branch of the cytochrome P450 arachidonic acid monooxygenases [38]. The arachidonic acid epoxygenases are expressed by endothelial cells both in vitro and in vivo [39–41] and catalyze the oxidation of arachidonic acid to four regioisomeric epoxyeicosatrienoic acids (EETs) [42, 43]. EETs have been shown to possess proangiogenic activities [39, 44–47] and we have demonstrated that WY14643-mediated PPAR*α* activation directly prevents endothelial cell migration and proliferation by downregulating endothelial arachidonate epoxygenase expression and EET biosynthesis [38]. Most importantly, in vivo treatment with WY14643 prevents primary tumor growth and tumor-associated angiogenesis by downregulating the levels of circulating EETs [38].

Consistent with the observation that PPAR*α* ligands might act as potent direct and/or indirect antiangiogenic factors, Panigrahy et al. have recently shown that fenofibrate suppresses VEGF-mediated endothelial cell proliferation as well as tumor cell-derived VEGF and FGF2 synthesis with concomitant stimulation of tumor-cells derived thrombospondin and endostatin [48]. Moreover, fenofibrate and WY14643 prevent VEGF-mediated endothelial cell migration by inhibiting Akt phosphorylation [24] and fenofibrate prevents endothelial cell proliferation by inhibiting cyclooxygenase-2 expression [25]. Finally, PPAR*α* agonists were found to inhibit endothelial VEGFR2 expression by preventing Sp1-dependent promoter binding and transactivation [23]. Some of the major PPAR*α* targets known to control endothelial cell functions and the effects of PPAR*α* ligands on angiogenesis are summarized in Figure 1 and Table 1.

In conclusions these studies strongly suggest that by preventing endothelial cell functions PPAR*α* ligands may protect the vasculature from pathological alterations associated with either metabolic disorders (i.e., atherosclerosis, diabetes) or cancer. Thus, PPAR*α* can be considered as a new class of “antiangiogenic” gene, and suggest that its ligands may function as effective antiangiogenic drugs.

## 4. PPAR*α* TARGETS IN CANCER

The observation that PPAR*α* is expressed by tumor cells [59–61] started studies of the role of this nuclear receptor and its ligands on the prevention of tumor cell proliferation in vitro and in vivo. In this context it has been shown that PPAR*α* ligands suppress the growth of several cancer lines—including colon, liver, breast, endometrial, and skin—in vitro [62–66], as we all inhibit the metastatic potential of melanoma cells in vitro and in vivo [67, 68]. Furthermore, PPAR*α* ligands decrease colon carcinogenesis [62] and the growth of human ovarian cancer in mice [49]. Although the mechanisms whereby PPAR*α* directly prevents tumor cell functions have not been investigated in details, potential targets have been identified. Clofibrate, a PPAR*α* ligand, significantly suppressed the growth of OVCAR-3 xenotransplanted tumors and inhibited ovarian tumor cell proliferation by increasing the expression of carbonyl reductase, an enzyme that promotes the conversion of protumorigenic prostaglandin E2 to inactive PGF2*α* [49]. Moreover, clofibrate reduced the levels of circulating VEGF in tumor-bearing mice [49], while bezafibrate, another PPAR*α* ligand, decreased the number of intestinal polyps in Apc^Min/+^ mice possibly by lowering serum level of triglycerides and upregulating lipoprotein lipase synthesis [27, 51]. Finally, PPAR*α* activation has been shown to inhibit vascular smooth muscle cell proliferation underlying intimal hyperplasia by inducing the expression of the tumor suppressor p16INK4a [56].

Whereas these studies clearly suggest that PPAR*α* activation might be beneficial in reducing cancer growth, studies from the Gonzales laboratory demonstrate that long-term administration of certain PPAR*α* agonists (clofibrate and WY14643) induces liver adenoma and carcinomas in rats and mice [35, 52, 69, 70]. The ability of PPAR*α* ligands to induce hepatocarcinoma is PPAR*α*-dependent and mediated by the novel microRNA let-7C/c-myc axis [52]. Let-7C is a micro RNA that controls cell growth by directly downregulating c-myc expression [52]. Upon treatment of mice with WY14643, the hepatic expression of let-7C decreases with the concomitant induction of c-myc and the increased expression of the oncogenic mir-17-92 cluster [52]. Thus, this novel rodent specific PPAR*α*-regulated pathway might be responsible for increased hepatocellular proliferation and tumorigenesis.

All together, these findings indicate that, with few exceptions, PPAR*α* ligands can be viewed as antitumorigenic agents either by directly preventing tumor cell functions or by preventing tumor-derived production of proangiogenic molecules. Some of the potential PPAR*α* targets that control tumor cell functions and the effects of PPAR*α* ligands on tumorigenesis are summarized in Figure 1 and Table 1.

## 5. PPAR*α* LIGANDS AND TUMORIGENESIS: LESSONS FROM MICE

The generation of PPAR*α* null mice has provided an excellent tool not only to determine whether the effects exerted by PPAR*α* ligand are indeed PPAR*α*-dependent, but also for discerning between host versus tumor-mediated PPAR*α* responses (see Table 2 for details). In this regard, we have shown that wild-type mice injected with isogenic PPAR*α* expressing tumor cells respond to WY14643 treatment and develop fewer and smaller tumors than untreated wild-type mice [38]. In contrast, the growth of the same tumor cells is not prevented in WY14643-treated PPAR*α* null mice [38]. In agreement with our finding, absence of PPAR*α* in the host animals abrogated the potent antitumor effect of fenofibrate [48]. Finally whereas in vivo activation of PPAR*α* prevents vascular smooth muscle cell proliferation underlying intimal hyperplasia, PPAR*α* deficiency leads to hyperplasia [56]. Taken together, these results strongly suggest that activation of PPAR*α* in the host is a key element in preventing unwanted pathological cell growth.

Although rodents are the only species in which activation of PPAR*α* promotes liver cancer, for a long time it was thought that Di(2-ethylhexyl)phthalate (DEHP), a commonly used industrial plasticizer, might cause liver tumorigenesis presumably via activation of PPAR*α* [55, 71]. The use of PPAR*α* null mice has disproved this idea, as this plasticizer is able to induce tumorigenesis in both wild-type and PPAR*α*-null mice [55, 71]. These results suggest the existence of pathways for DEHP-induced hepatic tumorigenesis that are independent of PPAR*α*, but most likely dependent on DEHP-mediated oxidative stress [55].

PPAR*α* null mice have been also instrumental to determine the role of rodent versus human PPAR*α* in the promotion of liver carcinogenesis. Morimura et al. have generated a PPAR*α*-humanized mouse in which the human PPAR*α* is expressed in liver under control of the Tet-OFF system. Interestingly, prolonged exposure to WY14643 in these mice only led to a 5% incidence of liver tumors—including hepatocellular carcinoma—compared to the 71% observed in mice expressing the mouse PPAR*α* [53]. More recently, Yanget al. generated a PPAR*α*-humanized transgenic mouse where the complete human PPAR*α* gene was introduced onto a PPAR*α*-null background [54]. These PPAR*α*-humanized mice express the human PPAR*α* in liver as well as other tissues and respond to fenofibrate treatment by lowering serum triglycerides and by inducing the expression of enzymes involved in fatty acid metabolism [54]. However, in contrast to wild-type mice, treatment with fenofibrate did not cause significant hepatomegaly, hepatocyte proliferation, and most importantly hepatocarcinoma [54]. Thus, this study shows that the protumorigenic let-7C/c-myc pathway is activated only by the rodent, but not the human PPAR*α* receptor. Most importantly, this work highlights the possibility that PPAR*α* ligands might be used as safe drugs for the treatment of cancer in humans.

Although activation of PPAR*α* in either endothelial or tumor cells has been proven to be beneficial in inhibiting cancer growth, it has also been shown that loss of host-derived PPAR*α* can be advantageous as it prevents tumor growth and development [57]. The host cells responsible for this protection, however, are granulocytes rather than endothelial cells. Loss of PPAR*α* leads to an increased infiltration to the side of injury of granulocytes that suppress tumor-associated angiogenesis via excess production of the endogenous angiogenesis inhibitor thrombospondin [57]. This study clearly indicates that both activation of PPAR*α* in specific host cells (i.e., endothelia cells) and concomitant inhibition of PPAR*α* in immuno cells (i.e., granulocytes) might lead to the same effects, namely protection from tumor growth.

## 6. CONCLUSIONS

The studies summarized in this review identify PPAR*α* as a potential host-based target for the development of new antiangiogenic approaches to inhibit and/or prevent tumor growth. As an established modulator of gene transcription, PPAR*α* regulates the expression of genes known to be involved in energy metabolism, cellular proliferation, and angiogenesis and to have positive effects on the control of dyslipidemia, inflammation, and cardiovascular diseases. Furthermore, several fibric acid derivatives bind to and activate human PPAR*α* with limited or no documented unwanted consequences and have proven to be safe and effective hypolipidemic drugs. In this context, gemfibrozil safely reduced the risk of death from coronary heart disease, nonfatal myocardial infarction, or stroke by raising HDL cholesterol levels and lowering levels of triglycerides [72, 73].

The effects of PPAR*α* ligands in animal models of tumor angiogenesis should help not only to stimulate further research of their usefulness as antitumorigenic agents, but also to facilitate their evaluation as valid tools for the treatment and/or prevention of human cancers. In this context, it is our hope that these studies will serve to encourage epidemiological studies of cancer incidence in patients using hypolipidemic drugs, and help to identify their potential beneficial effects as agents for tumor prevention and/or treatment. The urgency of new approaches for cancer treatment are indicated by the fact that most current antitumorigenic therapies are oriented towards a general inhibition of tumor cell growth and, as such, they suffer from lacking target selectivity and, in most cases, causing severe side effects and overall systemic toxicity. Thus, targeting PPAR*α* may prove to be a potential therapeutic strategy—either alone or in combination with conventional chemotherapy—to inhibit and ideally prevent cancer with excellent tolerance and limited toxicity.

## Figures and Tables

**Table 1:** Effect of PPAR*α* activation on angiogenesis and tumorigenesis.

Ligand	Cell type	Effect	Target	Reference
WY14643	Endothelial cells	Inhibition of cell proliferation and tubulogenesis in vitro Antiangiogenic activity in vivo	Downregulation of arachidonate epoxygenase synthesis	[38]

WY14643	Endothelial cells	Enhanced endothelial tube formation in vitro Proangiogenic activity in vivo	Upregulation of VEGF production	[37]

Fenofibrate WY14643* * * * ETYA	Endothelial cells	Inhibition of VEGF- or FGF2-mediated cell proliferation in vitro Antiangiogenic activity in vivo	Downregulation of VEGF production Upregulation of thrombospondin and endostatin production	[48]

Fenofibrate WY14643	Endothelial cells	Reduced cell migration	Inhibition of Akt activation	[24]

Fenofibrate	Endothelial cells	Reduced cell proliferation	Inhibition of cyclooxygenase-2 expression	[25]

Fenofibrate	Endothelial cells	Reduced cell proliferation	Inhibition of VEGFR2 expression	[23]

Clofibrate	Ovarian cancer cells	Reduced cell proliferation in vitro Antitumorigenic activity in vivo	Reduced prostanoid and VEGF levels via upregulation of carbonyl reductase expression	[49]

Methylclofenapate	Colonic adenocarcinoma	Reduced cell proliferation	Not investigated	[50]

Methylclofenapate	Apc^Min/+^ mice	Reduced number of intestinal polyps	Not investigated	[50]

Bezafibrate	APC^1309^mice Apc^Min/+^ mice	Reduced number of intestinal polyps	Reduced serum level of triglycerides and increased lipoprotein lipase synthesis	[27, 51]

WY14643	Wild-type mice	Enhanced hepatocellular proliferation and tumorigenesis in vivo	Downregulation of the miRNA let-7C with increased c-myc expression	[52]

**Table 2:** PPAR*α* and tumorigenesis: lessons from the PPAR*α*-null mice.

Ligand	Host	Challenge	Effect	Target	Reference
WY14643	PPAR*α*-null mice		Resistant to the development of spontaneous hepatocarcinoma	Inability to downregulate the miRNA let-7C	[52]

WY14643 Fenofibrate	PPAR*α*-humanized transgenic mouse		Resistant to the development of spontaneous hepatocarcinoma	Inability to downregulate the microRNA let-7C	[53, 54]

WY14643	PPAR*α*-null mice	Injection of isogenic tumor cells	Resistant to the Wyeth-mediated antiangiogenic and antitumorigenic activities	Inability to downregulate arachidonate epoxygenase expression	[38]

DEHP	PPAR*α*-null mice		Development of hepatocarcinoma	Increased PPAR*α*-independent oxidative stress	[55]

WY14643 Fenofibrate	PPAR*α*-null mice	Carotid arterial injury	Intimal hyperplasia	Inability to induce the expression of the tumor suppressor p16INK4a	[56]

	PPAR*α*-null mice	Injection of isogenic tumor cells	Resistant to the development of primary and metastatic tumor growth	Increased recruitment of granulocyte responsible for thrombospondin production	[57]

	PPAR*α*-null mice		Increased susceptibility to spontaneous adenomas and hepatocellular carcinomas	Not explored	[58]
